# A Case of New Onset Cervical Dystonia in Pregnancy

**DOI:** 10.5334/tohm.734

**Published:** 2022-12-23

**Authors:** Eoghan Donlon, Pat Moloney, Darragh Frier, Robert McFarlane, Shane Smyth, Fiona Molloy, Richard Walsh, Tim Lynch, Eavan McGovern

**Affiliations:** 1 Department of Neurology, Beaumont hospital, Dublin, IE; 2 Dublin Neurological institute, Mater Misericordiae hospital, Dublin, IE; 3 Department of Neurology Tallaght University Hospital, Dublin, IE; 4 School of Medicine, Royal College of Surgeons in Ireland, Dublin, IE

**Keywords:** Dystonia, Dystonia gravidarum, pregnancy, botulinum toxin

## Abstract

**Background:**

Though uncommon, primary movement disorders can occur in pregnancy, the most common being restless legs syndrome and chorea gravidarum [1]. New onset dystonia in pregnancy has been reported four times previously with a resolution of symptoms within six months of delivery [2,3,4,5]. Exacerbation of pre-existing movement disorders and the onset of de novo movement disorders during pregnancy support the hypothesis that female sex hormones play an important role in the regulation of basal ganglia circuitry.

**Case Report:**

Here we describe a case of new-onset cervical dystonia during pregnancy with persistence of symptoms after delivery.

**Discussion:**

The phenotypic overlap between this case and previously reported cases further establishes dystonia gravidarum as a distinct clinical entity.

## Case report

A 36-year-old right-handed, secundigravid, Caucasian woman presented at 8 weeks gestation with involuntary movements of the head, neck and right shoulder. Past medical history was significant for anxiety, hyperemesis gravidarum and drug-induced dystonia during her first pregnancy with complete resolution. Medications included fluoxetine 20 mg once daily for anxiety and Pyridoxine/doxylamine 10 mg/10 mg once daily for hyperemesis gravidarum. There was no history of thyroid disease or rheumatic fever. There was a family history of blepharospasm in the patient’s mother. Neurological examination revealed right torticollis, laterocollis to the left, and right shoulder elevation (Supplementary Video 1). The left sternocleidomastoid (SCM) was hypertrophied. There was no distractibility or entrainment. Touching the right side of chin relieved symptoms and supported the presence of a geste antagoniste. The remainder of the neurological examination was normal. Laboratory investigations for thyrotoxicosis, Wilson’s disease and autoimmune disorders were normal. A dystonia genetic panel identified a variant of unknown significance in the *ANO3* gene (c.2141+1G>A;p?) which is associated with DYT24. Segregation analysis identified the variant in unaffected family members, and it was, therefore, felt unlikely to be pathogenic. Treatment was initiated with procyclidine and clonazepam resulting in mild symptomatic improvement. Symptoms persisted throughout pregnancy and resolved completely 3 days post-partum. The patient began breastfeeding and once mature milk was produced the dystonic movements remerged, albeit less severe than during pregnancy. Botulinum toxin was injected in to the left SCM, right splenius capitus muscle and right trapezius with good symptomatic improvement. Over three years of regular follow up, symptom fluctuations have abated and evolved into a typical cervical dystonia with good response to regular botulinum toxin injections.

## Discussion

This is the 5^th^ reported case of dystonia with onset during pregnancy. The similarities between the five cases (Supplementary Table 1) highlight an emerging clinical phenotype. Our case is the only case in the literature demonstrating a recrudescence of symptoms with breastfeeding, and persistence of symptoms chronically.

In the cases described, DG generally emerges in the first trimester with progression of severity of dystonia through second and third trimesters. Symptoms resolve between the third trimester and 12 months post-partum, with marked improvement after delivery and no reports of recurrence of symptoms. Our case followed this typical course with dramatic resolution of symptoms after delivery. Unique to our case was a recrudescence of symptoms 5 days after delivery, seemingly coinciding with the production of mature breast milk and persistence of symptoms thereafter. With regard to phenomenology, a cervical dystonia phenotype was common to all cases with associated shoulder elevation in our case and 3 of the other reported cases.

We postulate a predisposition to nigrostriatal dysfunction in our patient on two fronts: her personal history of drug-induced orobuccal dyskinesia and a maternal history of blepharospasm. Drug-induced dystonia in a prior pregnancy was reported in one other case [2]. A personal history of essential tremor was described in one case [4] and a family history in another [3] adding further weight to a genetic predisposition to DG. The hormonal milieu of pregnancy may have acted as the “second hit” after the genetic “first hit” in a predisposed individual.

The prompt improvement or resolution of symptoms after delivery strongly supports the hormonal milieu of pregnancy being the key driver of nigrostriatal dysfunction. High-dose estrogen results in initial suppression and later enhancement of dopaminergic activity in the striatum, suggesting it modulates basal ganglia function both directly and indirectly [6]. Estrogen and progesterone levels begin to fall soon after delivery and normalise within three to four months postpartum. The recrudescence of symptoms with breastfeeding in this case adds further complexity to the proposed hormonal hypothesis as progesterone and estrogen fall precipitously after delivery. It’s possible, though entirely theoretically, that increased levels of prolactin that accompany lactation may alter dopaminergic activity within the basal ganglia through negative feedback in predisposed individuals. New onset dystonia in pregnancy, which persists chronically has not been described in the literature.

A combination of clonazepam and procyclidine provided some symptomatic relief to our patient. Clonazepam has a category D rating in pregnancy, though it has not been shown to have an association with developmental abnormalities [7]. Similarly, procyclidine’s safety in pregnancy has not been established, though it has a category C rating and is frequently used to treat drug-induced dystonic reactions in pregnancy. Two other cases described used clonazepam to good symptomatic effect [2,4]. Botulinum toxin administration in the post-partum period provided symptomatic relief in our patient and one other case [4].

The cases described in the literature establish DG as a distinct clinical entity which should be considered in women presenting with a de novo movement disorder arising in pregnancy. This case is unique in that DG did not resolve after delivery, rather it evolved into persistent cervical dystonia. Further research is needed to elucidate the hormonal mechanisms driving basal ganglia dysfunction in these patients as well as the likelihood of recurrence in future pregnancies.

## Additional Files

The additional files for this article can be found as follows:

10.5334/tohm.734.s1 Supplementary Material Video 1. Description of phenomenology: Prominent torticollis to the right, with laterocollis to the left and subtle right shoulder elevation. Demonstration of Geste antagoniste with resting the right hand under the chin. No evidence of generalised dystonia or gait abnormality. Mild reduction in severity of symptoms is noted after treatment with clonazepam 5 mg and procyclidine 0.5 mg.

10.5334/tohm.734.s2 Supplementary Material Table 1. Review of existing Dystonia Gravidarum cases in the literature.
