# Supplementary material for: A Case of New Onset Cervical Dystonia in Pregnancy
Source: Tremor Other Hyperkinet Mov (N Y). 2022 Dec 23;12:33. doi: 10.5334/tohm.734 (PMC9783953; doi:10.5334/tohm.734)
Supplement: Supplementary Material Table 1. — Review of existing Dystonia Gravidarum cases in the literature. [file tohm-12-1-734-s2.pdf]

Table 1. Review of existing Dystonia Gravidarum cases in the literature

	Presentation and past medical history (PMH)	Phenotype	Outcome
**Lim *et al.* (2006)**	31 year old primigravid<br>Symptom onset: 1st trimester (4/40))<br>PMH: Drug induced oculogyric crisis (prochlorperazine)	Torticlis to right, laterocolis to right, right shoulder elevation, head tremor. Left SCM hypertrophy. Geste antagoniste.	Reduced severity of symptoms through 2nd trimester with complete resolution in 3rd trimester.
**Fasano *et al.* (2007)**	28 year old, primigravid<br>Symptom onset: 1st trimester (1/40)<br>PMH: Likely ET<br>FH: 2 sisters ET	Torticollis to right, laterocolis to left, left shoulder elevation. Left SCM hypertrophy, blepharospasm, postural tremor. Geste antagoniste.	Resolved 3 months after delivery
**Buccoliero *et al.* (2007)**	28 year old, primigravid<br>Symptom onset: 1st trimester (4/40)<br>PMH:ET, hypothyroidism	Torticolis to right, anterocollis, postural and intention tremor.	Marked reduction in severity after delivery, complete resolution in 1 year.
**Madu (2013)**	30 year old, primigravid,<br>Symptom onset: 2nd trimester (17/40)	Anterocollis and retrocollis, left sholder elevation. Geste antagoniste.	Resolved completely 6 weeks after delivery.
**Current case**	36 year-old, secundigravid<br>Symptom onset: 1st trimester (8/40)<br>PMH: Drug induced dystonic reaction (Prochloperazine)<br>FH: mother blepharospasm	Torticollis to right, laterocollis to the left, and right shoulder elevation. Left SCM hypertrophy. Geste antagoniste.	Initial resolution of symptoms after delivery with recrudescence in 5 days and persistence of symptoms chronically
